# Real-Time Monitoring with a Wristwatch-Type Sodium- and Potassium-Ion Sensor Using a Screen-Printed Liquid-Junction Reference Electrode

**DOI:** 10.1021/acsomega.6c04240

**Published:** 2026-09-21

**Authors:** Isao Shitanda, Takuma Fujisawa, Noya Loew, Ryo Shirai, Yuta Imade, Yuri Okamoto, Shinya Yanagita, Hikari Watanabe, Masayuki Itagaki

**Affiliations:** † Department of Pure and Applied Chemistry, Faculty of Science and Technology, 26413 Tokyo University of Science, 2641 Yamazaki, Noda, Chiba 278-8510, Japan; ‡ Research Institute for Science and Technology, Tokyo University of Science, 2641 Yamazaki, Noda, Chiba 278-8510, Japan; § Department of Informatics, 12918 Kyoto University, 36 Yoshida-honmachi, Sakyo-ku, Kyoto 606-8501, Japan; ∥ Institute of Arts and Sciences, Tokyo University of Science, 2641 Yamazaki, Noda, Chiba 278-8510, Japan

## Abstract

Wearable ion sensors have gained increasing attention for the continuous noninvasive monitoring of ions in body fluids such as sweat. However, printed reference electrodes commonly used in potentiometric ion sensors require prolonged conditioning to achieve stable potentials, which limits practical wearable applications. Here, we integrated a screen-printed liquid-junction reference-electrode architecture with dual Na^+^- and K^+^-selective electrodes in a wearable wristwatch-type sensing platform. The liquid-junction reference becomes operable without the prolonged preconditioning required by poly­(vinyl butyral) (PVB)-type printed references. The sensor exhibited sensitivities of 48.3 mV decade^–1^ for Na^+^ and 46.4 mV decade^–1^ for K^+^. The printed reference electrode drifted by approximately 6 mV over 3000 s against a commercial reference in 10 mM NaCl. A control reference electrode fabricated without the liquid-junction layer showed a substantially greater dependence on the external KCl concentration, supporting the functional role of the liquid-junction layer. A fabric layer and a superabsorbent-fiber (SAF) layer were incorporated as a physical barrier and a sample-handling structure. A wristwatch-type wireless device was designed and constructed for real-time data acquisition, wireless transmission, and local storage on an SD card. The complete system was deployed at an active construction site. During on-body testing with a volunteer construction worker, the system acquired Na^+^- and K^+^-responsive potentiometric signals in real time, which were converted to apparent ion concentrations using aqueous calibration curves, over a working period of approximately 2 h 45 min

## Graphical abstract

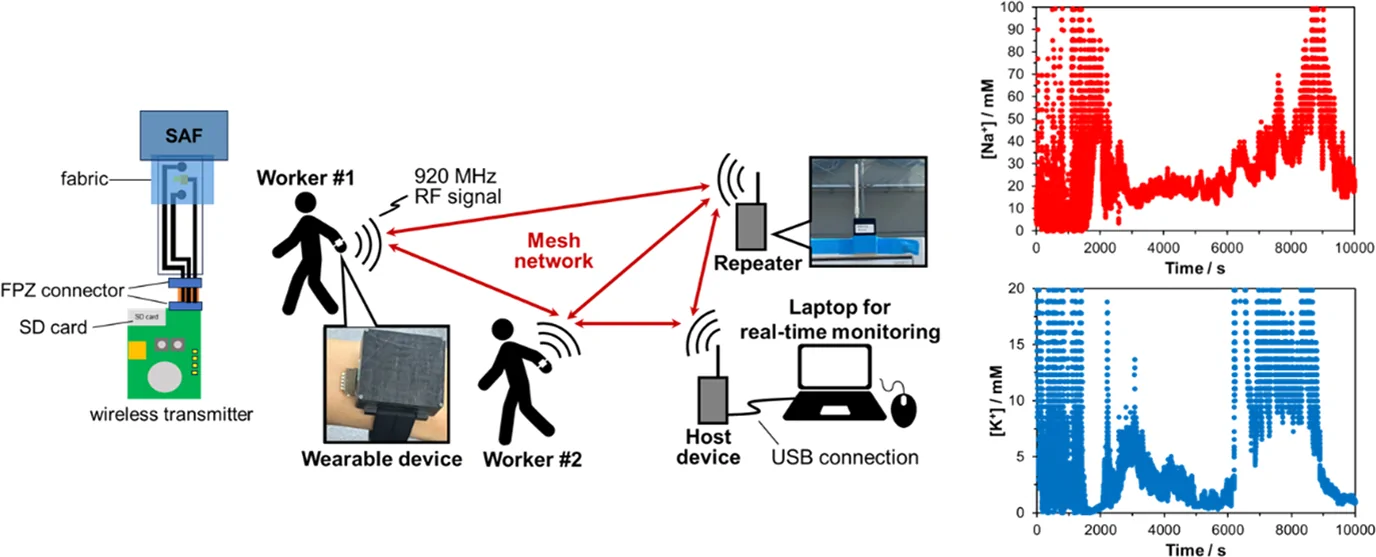

## Introduction

Wearable sensors that can be applied directly to the skin have recently attracted significant attention in the healthcare field.^1,2^ These sensors noninvasively or minimally invasively measure physical and chemical biomarkers such as heart rate, respiratory rate, and specific components of bodily fluids, representing a promising complementary technology to conventional vital-sign measurements.^3–5^ Wearable sensor systems have become practical through innovations in hardware and communication technologies, including advances in electronic-device miniaturization and weight reduction, as well as the widespread adoption of internet communication infrastructure. Wearable sensors enable the continuous monitoring of vital signs and biomarkers in real time during daily activities without requiring interruptions for special procedures.

Wearable chemical sensors and biosensors have been developed for the noninvasive acquisition of biochemical information from bodily fluids, including sodium, potassium, chloride, glucose, and lactate concentrations. Sweat,^6–9^ urine,^10^ saliva,^11^ and tears^12^ are target bodily fluids used as sources of biochemical information. Among these, sweat is easy to collect and amenable to continuous monitoring.

Ion sensors are particularly suitable for miniaturization owing to their potentiometric measurement principles and, consequently, their size-independent responses. Furthermore, monitoring ion concentrations in sweat in real time may provide information relevant to hydration status and heat-related health management. Therefore, ion sensors comprising an ion-selective electrode (ISE) and a reference electrode (RE) are popular components of wearable sensing devices. Gao et al. fabricated an ion sensor capable of simultaneously monitoring sodium, potassium, and lactate using sputtering.^13^ Yang et al. prepared an ion sensor for the simultaneous measurement of sodium- and potassium-ion levels using screen printing,^14^ while Shitanda et al. fabricated ion sensors capable of detecting sodium-, potassium-, ammonium-, and chloride-ion concentrations using screen and transfer printing.^15^ A variety of other wearable ion sensors have also been reported.^7–9^

Ion sensors are often fabricated using printing technologies owing to their distinct advantages, including process simplicity, high design flexibility, and mass-production scalability. In particular, printed sensors can be produced at low cost and are therefore suitable for disposable devices, which addresses issues associated with hygiene and adhesiveness, further highlighting the practicability of printed sensors.

While the performance of a potentiometric ion sensor depends on the characteristics of both the ISE and RE,^16^ most reports on ion sensors have focused on the ISE. The RE of an ion sensor is expected to exhibit a stable potential immediately following the commencement of analysis and maintain that potential for the duration of use. The RE in a printed ion sensor is often fabricated as a polyvinyl butyral (PVB) film containing electrolytes such as KCl coated over a Ag/AgCl electrode, a method first reported by Guinovart et al.^17^ More recently, Kuczak et al. reported printable REs using ionic liquids,^18^ and Malek et al. reported a printable solid-state Si/Ag/AgCl/KCl RE.^19^ However, while these REs maintain stable potentials, a significant amount of time is required for their initial stabilization, which constitutes a major barrier to practical applications because conditioning is required prior to use.

Komoda et al. developed a printable liquid-junction RE using silica gel and poly­(vinylidene fluoride-*co*-hexafluoropropylene) (PVDF–HFP), which stabilized rapidly following immersion in the measurement solution.^20^ The hydrophilicity of silica gel enables water to readily permeate into the KCl-containing electrolyte layer, thereby enabling rapid initial stabilization.

In this study, we incorporate a screen-printed liquid-junction reference electrode, adapted from the design of Komoda et al.,^20^ into a wearable dual-ion sensor together with Na^+^- and K^+^-selective electrodes, a fabric/superabsorbent-fiber (SAF) sample-handling layer, and a custom wristwatch-type wireless unit. The contribution of this work is not a new electrode material but the integration of these components into a single device that is operable without prolonged preconditioning and its operation under real occupational field conditions. We report bench characterization of the reference electrode, the two ion-selective electrodes, and their single-salt cross-response behavior, together with the sample-transport behavior of the fabric/SAF configuration, followed by a field-feasibility demonstration at an active construction site. We do not claim fully validated quantitative sweat analysis; the on-body results are reported as Na^+^- and K^+^-responsive potentiometric signals converted to apparent concentrations using aqueous calibration curves.

## Materials and Methods

### Materials

*N*,*N*′-Dimethylpropyleneurea (DMPU), Triton X-100, potassium chloride (KCl), isophorone, silver nitrate (AgNO_3_), polyvinyl chloride (PVC), bis­(2-ethylhexyl) decanedioate (dioctyl sebacate, DOS), and valinomycin were purchased from FUJIFILM Wako Pure Chemical Corporation. Poly­(vinylidene fluoride-*co*-hexafluoropropylene) (PVDF–HFP), silica gel, sodium ionophore X, sodium tetrakis­[3,5-bis­(trifluoromethyl)­phenyl]­borate (Na-TFPB), and potassium tetrakis­(4-chlorophenyl)­borate (KTClPB) were obtained from Sigma-Aldrich. Silver ink was purchased from Sanwa Chemical, and carbon ink (JELCON CH-10) was purchased from Jujo Chemical. Release-agent-treated polyethylene terephthalate (PET) films, overcoat ink, and adhesive ink were purchased from Japan Polymark Co., Ltd. Stretchable resist ink (XKZ10600W) was obtained from Namics Corporation. Superabsorbent fiber sheets (SAF; Oji Kinocross, 100 g m^–2^ fiber, 230 g m^–2^ absorbency) were obtained from Oji Holdings.

### Silver/Silver-Chloride Ink Preparation

Silver chloride (AgCl) was prepared by dissolving AgNO_3_ (20.0 g) in water and adding KCl (10.0 g). The resulting precipitate was collected by suction filtration, washed with deionized water and methanol, and then ultrasonically dispersed for 3 h to afford AgCl powder. Ag/AgCl ink was prepared by mixing the AgCl powder with commercial silver ink in a 1:10 (w/w) ratio.

### Liquid-Junction Ink Preparation

Triton X-100 (1.0 g), PVDF–HFP (1.0 g), silica gel (1.0 g), and isophorone (8.0 g) were mixed with a magnetic stirrer until dissolved, after which the mixture was dispersed using an ultrasonic disperser (Nano Taro; THINKY).

### Electrolyte Ink Preparation

Triton X-100 (1.0 g), PVDF–HFP (1.0 g), silica gel (1.0 g), and DMPU (8.0 g) were mixed with a magnetic stirrer until dissolved, after which KCl (4.0 g) was added and the mixture was dispersed for 3 h using the ultrasonic disperser.

### Fabricating Screen-Printed Electrodes

Electrodes were printed on release-agent-treated PET film using a screen printer (LS-150TV, Newlong Seimitsu Kogyo, Japan) (Figure 1a,b). Layers of overcoat and resist ink were sequentially printed and dried on the PET film; these layers provide heat protection during adhesion to cloth. Five layers of carbon ink were subsequently printed to form working electrodes and leads. Layers of the Ag/AgCl ink, electrolyte ink, and liquid-junction ink were then sequentially printed to form the RE. Finally, a layer of stretchable resist ink was printed to define the electrode areas, followed by a layer of adhesive ink to facilitate fabric attachment. The working-electrode area was 0.0707 cm^2^ and the reference-electrode area was 0.12 cm^2^. Printed-layer thicknesses were not measured.

**Figure 1:**
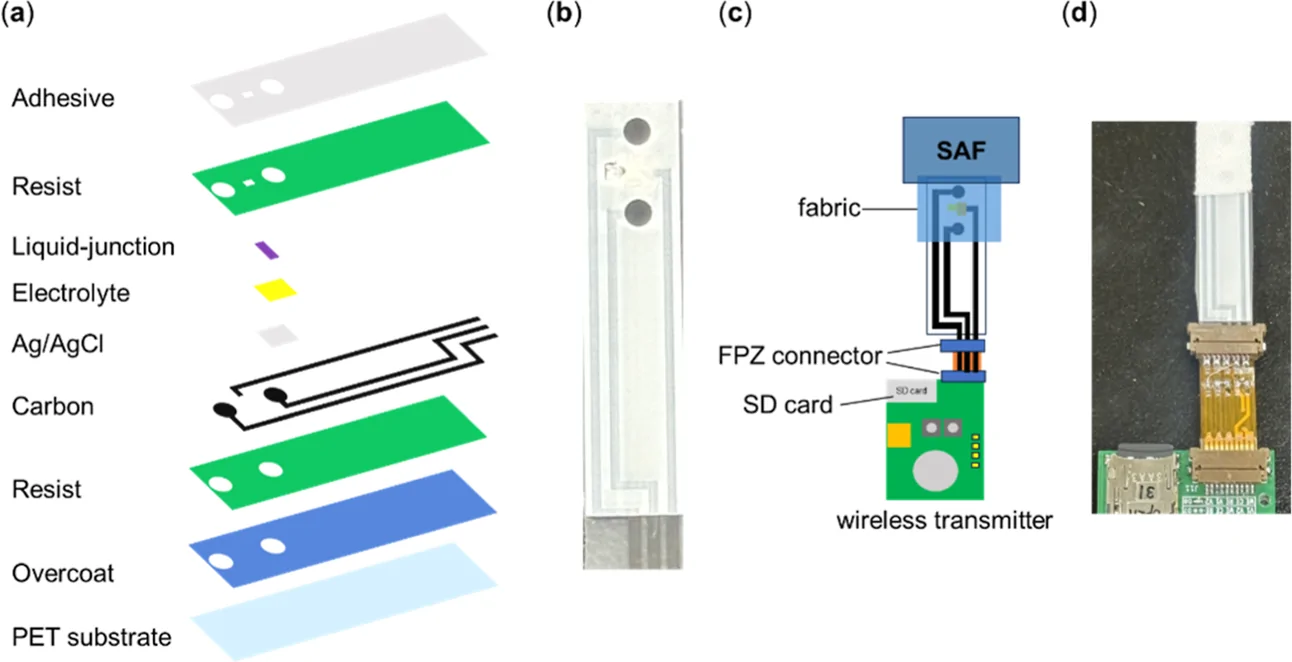
Screen-printed dual-ion sensor and wireless sensing device. (a) Schematic and (b) photographic image of the printed sensor. (c) Schematic and (d) photographic image of the wireless sensing device (sensor with fabric, FPZ connector, and partial wireless transmitter).

### Fabricating the Sodium-Ion-Selective Electrode

Sodium ionophore X (2 mg), Na-TFPB (0.5 mg), PVC (33 mg), and DOS (65.5 mg) were dissolved in tetrahydrofuran (THF, 660 μL) and the solution was ultrasonicated for 30 min. A 1 μL drop of the solution was then deposited on one of the carbon working electrodes (WEs) and dried overnight to form the Na^+^-selective membrane.

### Fabricating the Potassium-Ion-Selective Electrode

Valinomycin (2 mg), KTClPB (0.5 mg), PVC (33 mg), and DOS (65.5 mg) were dissolved in THF (1 mL) and the solution was ultrasonicated for 30 min. A 1 μL drop of the solution was then deposited on the other carbon working electrode and dried overnight to form the K^+^-selective membrane.

### Assembling the Wireless Dual-Ion Sensing Device

A piece of fabric was attached to the electrodes of the sensor strip using heat-transfer printing (Figure 1c,d). The fabric was pressed to the electrode at 110 °C and 0.8 MPa for 10 s using a Pony 50 transfer printer (Japan Polymark); the adhesive layer melted and bonded to the fabric under these conditions. The fabric served as a physical barrier between the printed electrode and the skin and was intended to promote wetting of the sensing area. A piece of SAF was then attached to the end of the fabric away from the electrode to promote sample transport away from the sensing area. The dual-ion sensing device was completed by connecting the sensor to a custom-made wristwatch-type wireless transmitter via an FPZ connector (Figure 1c,d). The transmitter was also equipped with a micro-SD card for data storage. The fabric measured 2.5 mm × 10.5 mm and the SAF measured 5.0 mm × 21 mm, with a sensor-to-SAF distance of approximately 5 mm.

### Electrochemical Evaluation

A potentiostat (EmStat, PalmSens Co., Ltd.) was used to evaluate the sensor electrodes without the SAF attached. The printed liquid-junction RE was evaluated against a commercial Ag/AgCl/saturated-KCl reference electrode (BAS). The ion-selective electrodes were calibrated against the same commercial Ag/AgCl/saturated-KCl reference electrode, consistent with the potential axes in Figures 3 and 4. Open-circuit potentials (OCPs) were measured using electrodes immersed in aqueous solutions containing various concentrations of NaCl or KCl. The short-term stability of the printed reference electrode was additionally evaluated in 10 mM NaCl (*n* = 3 independently fabricated electrodes). As a functional control, a printed reference electrode was fabricated using the same process as the liquid-junction reference electrode, including the KCl-containing electrolyte layer, except that the liquid-junction layer was omitted. The control electrode was evaluated against the commercial Ag/AgCl/saturated-KCl reference electrode in 1 and 100 mM aqueous KCl for 3000 s (Figure S3). Single-salt cross-response behavior was evaluated by the separate-solution method against the commercial Ag/AgCl/saturated-KCl reference electrode. Each ion-selective electrode was immersed in single-salt NaCl and KCl solutions at 1, 10, 100, and 1000 mM. Apparent concentration-based coefficients were estimated from the potentials at 10 mM using the theoretical Nernstian slope of 59.2 mV decade^–1^.

**Figure 2:**
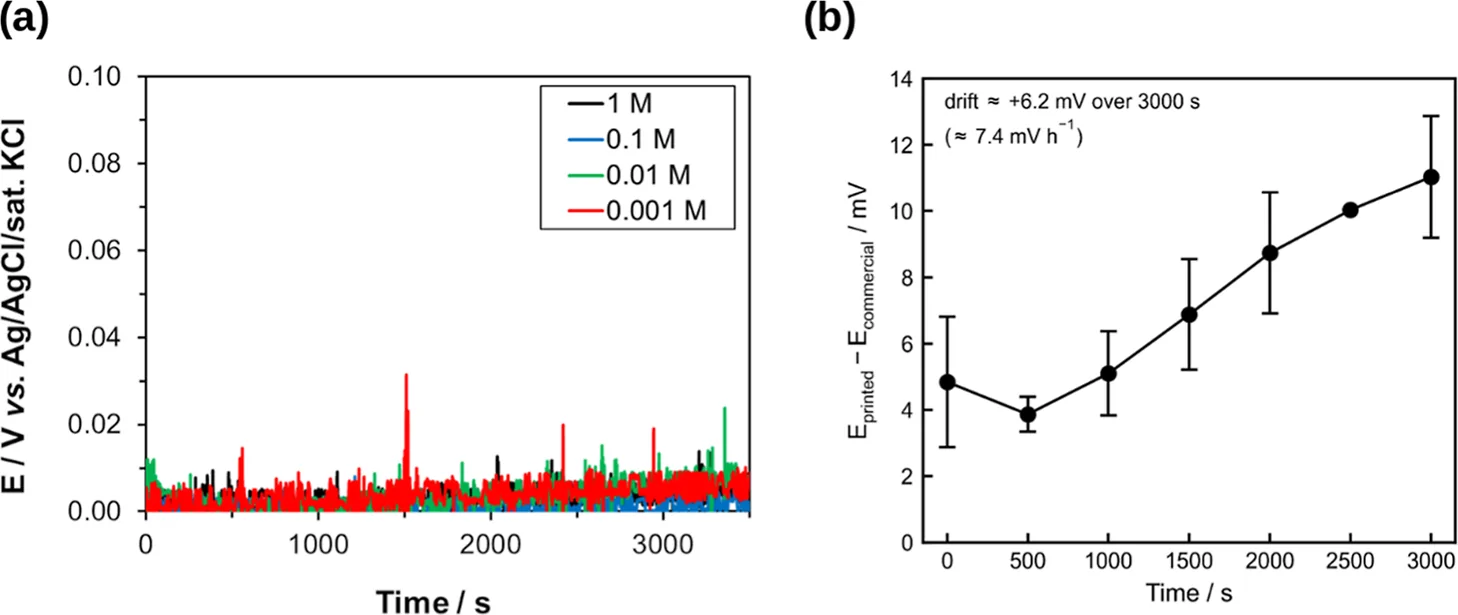
Evaluation of the integrated liquid-junction reference electrode. (a) Potential difference between the printed liquid-junction and the commercial Ag/AgCl/saturated-KCl reference electrodes as a function of time, measured immediately after immersion in aqueous NaCl at several concentrations. (b) Potential difference in 10 mM NaCl; symbols are means and error bars represent the standard deviation (*n* = 3 independently fabricated electrodes). The transfer-printed fabric was present and the separate SAF was absent.

**Figure 3:**
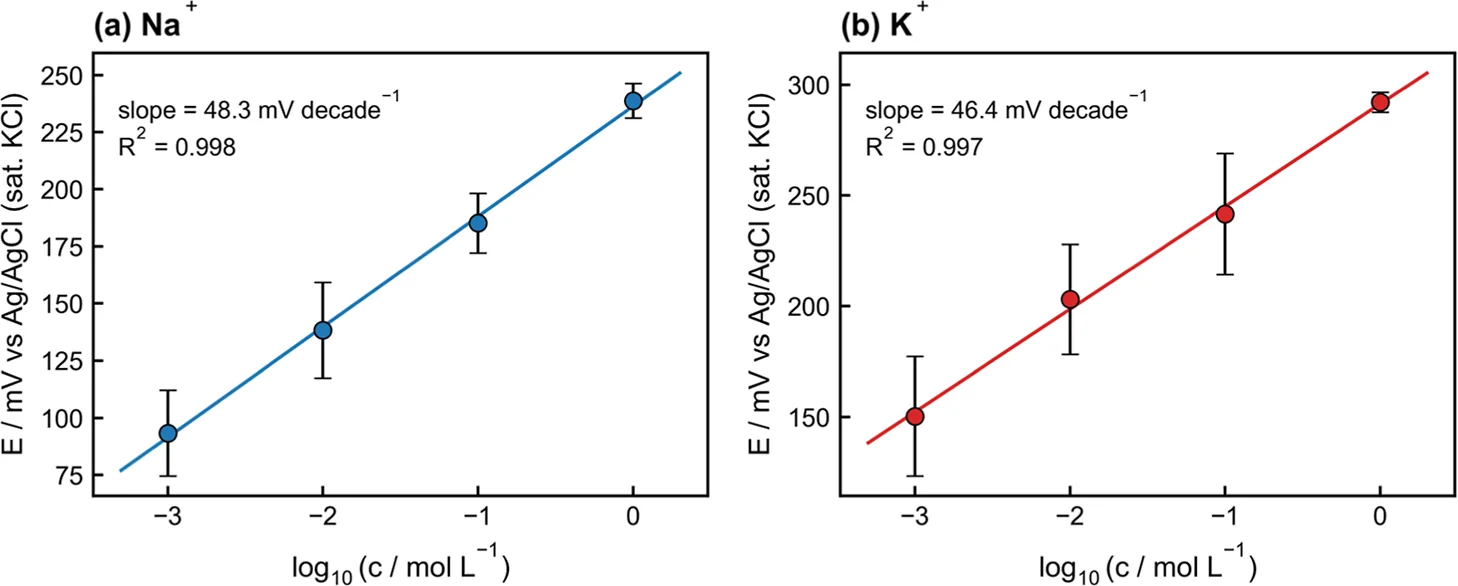
Calibration of the (a) sodium-ion and (b) potassium-ion sensors, measured against the commercial Ag/AgCl/saturated-KCl reference electrode. Symbols are means and error bars represent 95% confidence intervals (*n* = 3 independently fabricated electrodes).

**Figure 4:**
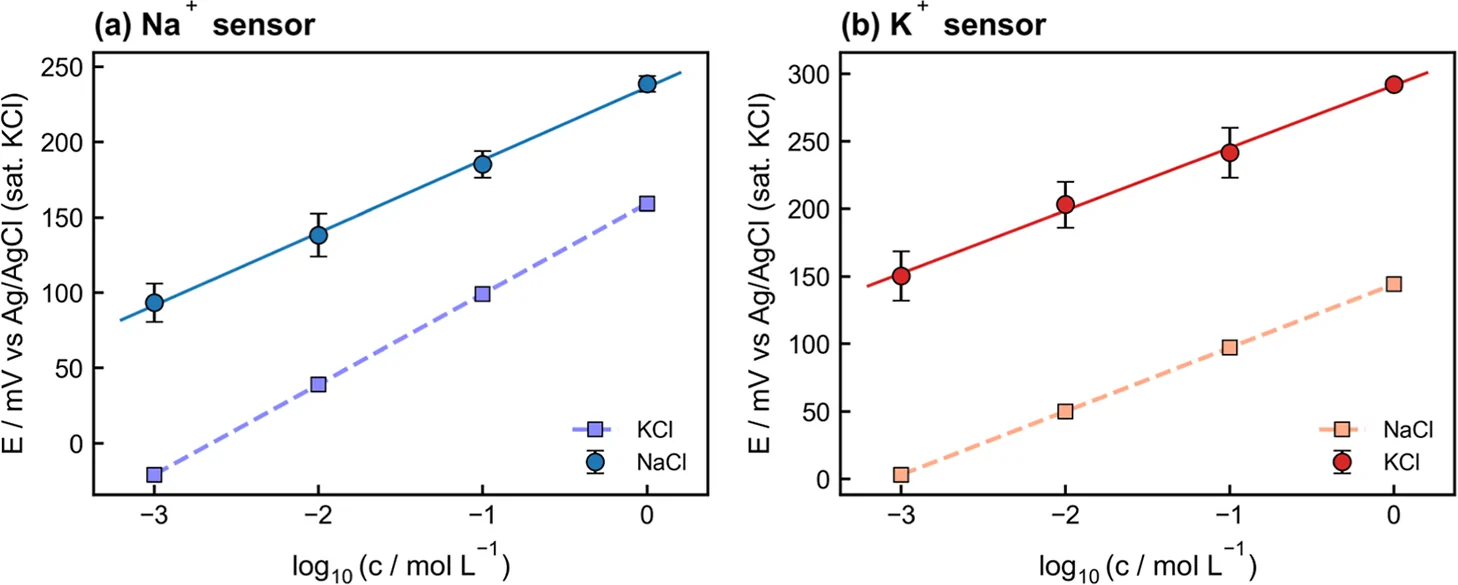
Single-salt cross-response evaluation of the (a) sodium-ion and (b) potassium-ion sensors by the separate-solution method, measured against the commercial Ag/AgCl/saturated-KCl reference electrode. Open-circuit potential versus log_10_(*c*/mol L^–1^) in single-salt NaCl and KCl solutions (1, 10, 100, and 1000 mM). Error bars represent 95% confidence intervals (*n* = 3 independently fabricated electrodes).

### Evaluating Flow

Flow (i.e., old/new sample exchange) was evaluated with the SAF attached. Aqueous NaCl solutions of various concentrations were dropped onto the sensor fabric at a constant flow rate using a syringe pump. The response of the Na sensor was monitored using a potentiostat, and the relationship between sensor response and NaCl concentration was evaluated. The solutions were supplied at 10 μL min^–1^, with each concentration applied for 300 s. Thus, 50 μL of each solution was delivered at each concentration. The flow rate was selected for controlled bench testing and was not intended to reproduce a physiological sweat rate. No microfluidic channel was used, so the local flow was not strictly confined, and sample loss and evaporation were not independently controlled.

### On-Body Testing

On-body testing was conducted at an active construction site managed by Sanwa Kensetsu Co., Ltd. One construction-worker volunteer was asked to wear one wristwatch-type wireless sensing device while performing their assigned tasks according to their usual routines, including breaks, hydration, and meals. Testing was conducted in accordance with ethical guidelines approved through the “Medical Research Plan Involving Human Subjects” review process of the Tokyo University of Science. The recorded potentials were converted to apparent Na^+^ and K^+^ concentrations using the aqueous calibration curves of Figure 3. No independent sweat sampling and no pre- or postfield calibration were performed, and the calibration conditions (immersion, fabric present, SAF absent, controlled temperature, laboratory electronics) differ from the on-body conditions.

## Results and Discussion

### Integrated Printed Liquid-Junction RE

The integrated liquid-junction RE was evaluated against a commercial Ag/AgCl/saturated-KCl reference electrode (Figure 2a). The printed liquid-junction RE did not exhibit the prolonged initial transient observed for the PVB-type printed reference electrode (Figure S1); however, a formal stabilization time was not determined using a predefined criterion. Here, “operable without prolonged preconditioning” means that potentiometric measurement could be initiated without the long initial transient observed for the PVB-type printed reference electrode; it does not imply that the reference potential had reached a fully stabilized value immediately after immersion. Its potential remained close to that of the commercial reference, with a residual difference that was small but not zero and that was largely independent of the Cl^–^ concentration of the solution over the range tested. In 10 mM NaCl (*n* = 3 independently fabricated electrodes), the printed-minus-commercial potential difference increased from about 5 mV to about 11 mV over 3000 s, corresponding to a drift of about 6 mV over 3000 s (about 7 mV h^–1^) (Figure 2b). This drift is small but not negligible relative to the approximately 48 mV decade^–1^ sensitivity of the ion-selective electrodes and may contribute uncertainty to the field measurements. Transient fluctuations were observed in the printed-RE potential, but their origin was not determined. In comparison, a PVB-type printed reference electrode required more than 1000 s to stabilize when measured from immersion in 0.1 M NaCl (Figure S1). As a functional control, a printed reference electrode fabricated without the liquid-junction layer was evaluated against the commercial Ag/AgCl/saturated-KCl reference electrode in 1 and 100 mM KCl (Figure S3). The potential of the control electrode depended strongly on the external KCl concentration, differing by approximately 80 mV between 1 and 100 mM KCl at 3000 s, and continued to change with time over the 3000 s measurement. In contrast, the potential of the liquid-junction reference electrode was largely independent of the external Cl^–^ concentration over the NaCl series in Figure 2a. Because the control electrode was evaluated in KCl whereas the data in Figure 2 were acquired in NaCl, the two data sets do not constitute a condition-matched quantitative comparison; nevertheless, the control result supports the functional role of the liquid-junction layer in reducing sample-composition-dependent shifts in the printed reference potential. Only the short-term behavior of the printed reference electrodes was assessed. The long-term stability of the complete Na^+^/K^+^ sensing system, including drift of the ion-selective electrodes, was not evaluated in this study.

### Evaluating the Printed Ion Sensors

The sodium- and potassium-ion sensors were evaluated individually (Figure 3). The sodium and potassium sensors exhibited sensitivities of 48.3 mV decade^–1^ for Na^+^ and 46.4 mV decade^–1^ for K^+^ (Figure 3), corresponding to approximately 82% and 78% of the theoretical Nernstian slope (59.2 mV decade^–1^ at 25 °C). The observed slopes were below the theoretical Nernstian value; the origin of this deviation was not determined in the present study.

The Na^+^- and K^+^-selective membranes follow the conventional plasticized-PVC ion-selective-electrode design. Sodium ionophore X and valinomycin provide recognition of Na^+^ and K^+^, respectively, while the lipophilic ionic additives (Na-TFPB and KTClPB) support the ion-exchange response and charge balance within the membrane. DOS plasticizes the PVC phase and permits ion mobility within the membrane, and the printed carbon layer serves as the solid contact between the ion-selective membrane and the electronic conductor.^15,16^ The present study did not independently isolate or optimize the contribution of each membrane component.

The variability was larger at the lower ion concentrations (Figure 3); the origin of this concentration-dependent variability was not investigated. The membrane composition was not optimized for the heat-pressed, fabric-attached format, which we note as a limitation. Error bars in Figure 3 represent 95% confidence intervals (*n* = 3 independently fabricated electrodes).

### Selectivity of the Printed Ion Sensors

The two membranes were evaluated by the separate-solution method in single-salt NaCl and KCl solutions against the commercial Ag/AgCl/saturated-KCl reference electrode (Figure 4). Apparent concentration-based separate-solution coefficients were estimated from the open-circuit potentials at 10 mM using the Nicolsky–Eisenman relation with the theoretical Nernstian slope of 59.2 mV decade^–1^, giving log *K* ^pot^_Na,K_ ≈ −1.7 for the sodium sensor and log *K* ^pot^_K,Na_ ≈ −2.6 for the potassium sensor. Because the measured primary-ion slopes were sub-Nernstian, these values provide an approximate cross-response index rather than rigorous thermodynamic selectivity coefficients. Under the tested single-salt conditions, the K^+^-selective electrode showed a substantially smaller response to NaCl than to KCl, whereas the Na^+^-selective electrode exhibited an appreciable response to KCl. With the apparent *K* ^pot^_Na,K_ value of approximately 0.02, the equivalent contribution of K^+^ to the apparent Na^+^ concentration is approximately 2% of the K^+^ concentration under the concentration-based Nicolsky–Eisenman approximation for monovalent ions; thus, when the K^+^/Na^+^ concentration ratio is substantially below unity, the corresponding bias in the apparent Na^+^ concentration is expected to be small relative to the Na^+^ concentration itself. The potentials were measured at 10 mM without activity correction; fixed-interference and mixed-ion validation were not performed.

### Evaluating Flow and Sample Exchange

We next evaluated the response of the sensor under SAF-assisted flow (Figure 5). The concentration of the test solution was first increased and then decreased, with the response of the sodium sensor used as an indicator of the sodium-ion concentration at the sensing area. The SAF-assisted configuration produced concentration-dependent responses that tracked the applied sample solution (Figure 5). The SAF was positioned at the distal end of the fabric with the intention of drawing liquid away from the sensing area. While the sensor did not always transition instantaneously to the new concentration, the response approached a plateau within each concentration interval. The increasing and decreasing sequences gave similar calibration curves under the tested conditions (Figure 5b).

**Figure 5:**
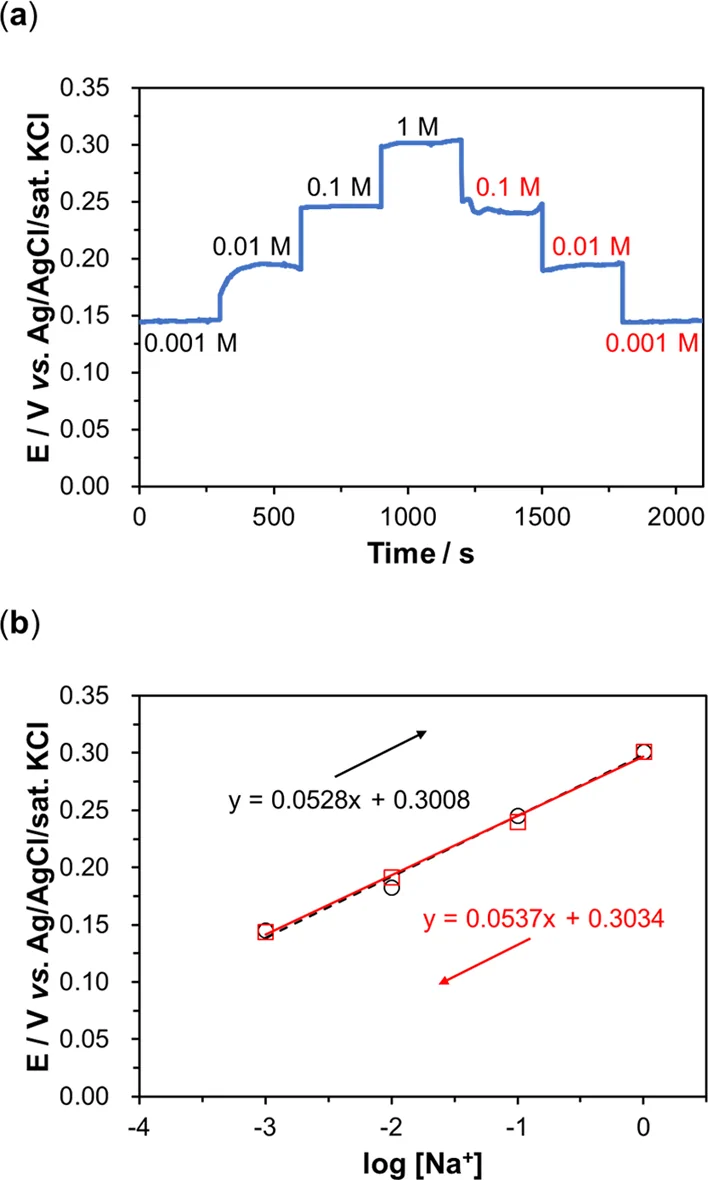
Response of the sodium-ion sensor during stepwise NaCl delivery through the fabric/SAF configuration at 10 μL min^–1^, with each concentration supplied for 300 s. Potentials were measured against a commercial Ag/AgCl/saturated-KCl reference electrode using a potentiostat. (a) Continuous response during increasing (black) and decreasing (red) concentration sequences. (b) Corresponding potential versus log_10_(*c*/mol L^–1^) plots.

The sodium-ion sensor gave higher potentials under SAF-assisted flow (Figure 5b) than when immersed without the SAF (Figure 3a). One possible explanation is preferential water removal by the SAF, which could concentrate the sample at the sensing area and shift the potential to higher values; this was not established experimentally. A known-concentration recovery test through the complete fabric/SAF structure was not performed, so the apparent concentrations obtained on-body cannot be assumed to equal the sweat composition at the skin surface; such a recovery test is a subject of future work.

### Wearable-Device and Measurement-Circuit Designs

We designed a wearable device with a printed circuit board (PCB) in a casing that can be fastened to the wrist (Figure 6). An FPZ connector, which is a type of zero-insertion force (ZIF) connector for use with flexible flat cables (FFCs), was used to connect the sensor to the PCB. The device is designed to be worn with the sensor under the casing to ensure close contact between the sensing area of the sensor and the skin.

**Figure 6:**
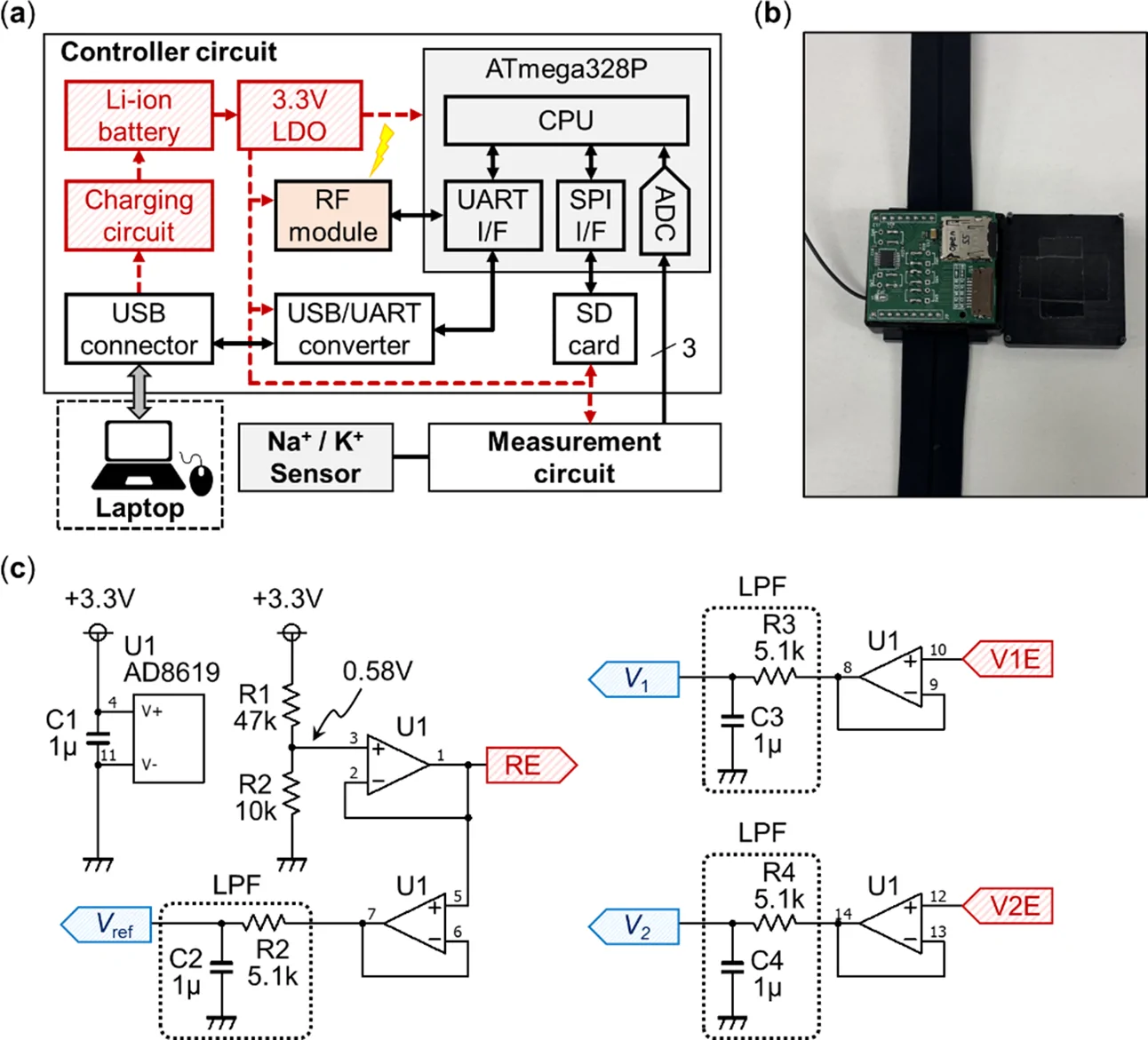
Schematic of the wristwatch-type wearable device. (a) Circuit architecture comprising the controller circuit, measurement circuit, sensor, and optional laptop. Solid black lines and dashed red lines indicate data flow and power distribution, respectively. (b) Photographic image of the circuit board in the wristwatch-type wearable device. (c) Measurement circuit with terminals for one RE and two WEs (V_1E_ and V_2E_).

The controller circuit employs an ATmega328P microcontroller (Microchip Technology Inc.) as the main processing unit (Figure 6a); this controller is responsible for communication with the wireless RF module, controlling the SD card, and analog-to-digital (A/D) conversion of the sensor signals received from the measurement circuit. A USB connector mounted on the controller circuit facilitates battery charging and wired sensor-signal acquisition; a laptop can be connected via USB for wired control or to configure the wearable device, but is disconnected when the device is worn by a subject. The measurement circuit acquires analog output signals from the ion sensors and delivers them to the controller circuit, where they are digitized via the ATmega328P on-chip analog-to-digital converter (ADC). The digitized data are transmitted to a host device with a laptop via an RF module and simultaneously recorded on a micro-SD card. The host device is placed within transmission range for real-time monitoring. The data stored on the micro-SD card can also be used for offline evaluation.

The ATmega328P on-chip ADC was operated at 10 bit resolution using the 3.3 V regulator output as the voltage reference. Before measurement, the regulator output was measured with a multimeter, and the measured value was used for voltage conversion; the voltage increment was approximately 3.223 mV per least significant bit (LSB) at the nominal 3.3 V reference. Sensor signals were sampled at 10 samples s^–1^ (0.1 s intervals). The analog low-pass filters had a cutoff frequency of 31.2 Hz. The ion-selective-electrode inputs were buffered by voltage followers to provide high input impedance; the input impedance was not independently measured. Each 0.1 s measurement record was first written to a 512 byte memory buffer and transferred to the micro-SD card when the buffer became full, so physical SD-card write operations occurred approximately every 2 s rather than after every sample. Wireless data packets were generated nominally every 5 s. If acknowledgment from the host was not received, transmission was retried after a network-dependent timeout. Because the system used a mesh network, the retry timing depended on network conditions, ranging from less than 1 s for promptly detected communication errors to more than 10 s when route discovery was required.

The measurement circuit provides three terminals (RE, V_1E_, and V_2E_), which are connected to the RE, K^+^-, and Na^+^-selective electrodes, respectively (Figure 6c). The RE-terminal voltage is set to approximately 0.58 V relative to the circuit ground using a voltage divider comprising resistors R1 and R2. This voltage facilitates symmetric measurement margins in both the positive and negative directions relative to the RE voltage. The divider output is buffered by a voltage follower before being supplied to the RE terminal to reduce the output impedance. The voltage at the RE terminal is further buffered and passed through a low-pass filter (LPF) before being output to the controller circuit as *V*_ref_. Similarly, the voltages of the K^+^- and Na^+^-selective electrodes relative to the circuit ground are each buffered using voltage followers to ensure high input impedances, filtered by LPFs, and then output to the controller circuit as *V*_1_ and *V*_2_. This circuit configuration attenuates high-frequency electronic noise. The controller circuit performs A/D conversion of *V*_ref_, *V*_1_, and *V*_2_ and calculates the differences *V*_1_ – *V*_ref_ and *V*_2_ – *V*_ref_; these voltage differences are transmitted and stored as the K^+^- and Na^+^-sensor responses. For a qualitative check, the Na^+^-sensor signal recorded with this circuit was compared with that recorded using a commercial potentiostat in 1 mM NaCl; the raw traces, with no data points removed, are provided in the Supporting Information (Figure S2). The two traces were recorded separately, and no quantitative agreement was established.

### Construction-Site Field Testing and Real-Time Monitoring System

We set up a real-time monitoring system at an active construction site to field-test our device (Figure 7). Repeaters were mounted at strategic points throughout the construction site, and a host device with a laptop was installed. One construction-worker volunteer was fitted with the wearable sensing device and asked to perform the assigned tasks according to the usual routine, including breaks, hydration, and meals. The wearable device established a mesh-network connection with the repeaters and the host device via wireless communication in the 920 MHz band. Repeaters and a host device were deployed to maintain wireless connectivity across the construction site, and the mesh-network configuration enabled transmission of the acquired signals to the host device during the field test, including when the worker wearing the sensing device moved within the obstructed environment.

**Figure 7:**
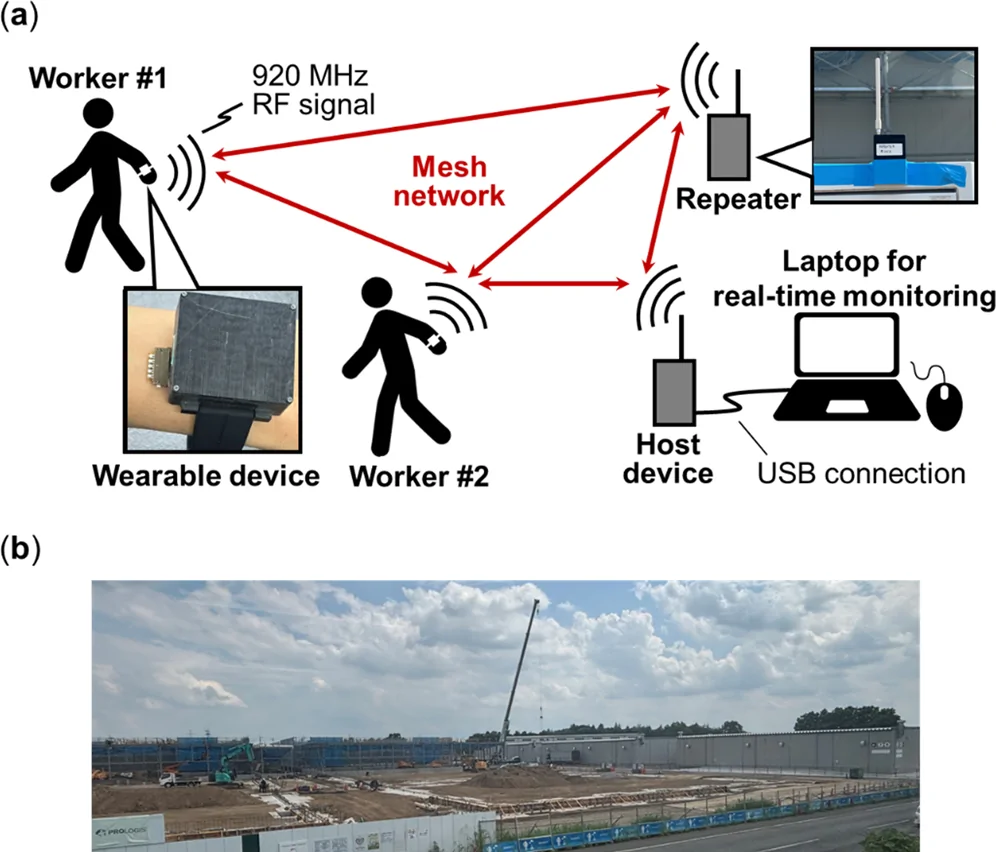
Real-time monitoring system at a construction site. (a) Schematic of the mesh-network architecture with repeater and host device; multiple worker nodes are shown schematically, although the present field test used one worker and one device. (b) Photographic image of the construction site.

### On-Body Testing at a Construction Site

On-body testing was conducted with one volunteer construction worker wearing one wristwatch-type device, and one useable data set was obtained. Figure 8 shows the data set obtained from this field test. The recorded potentials were converted into apparent Na^+^ and K^+^ concentrations using the aqueous calibration curves (Figure 8). The signal was unstable before approximately 2000 s, which may reflect incomplete wetting of the sensing area; the onset of sweating was not independently determined.

**Figure 8:**
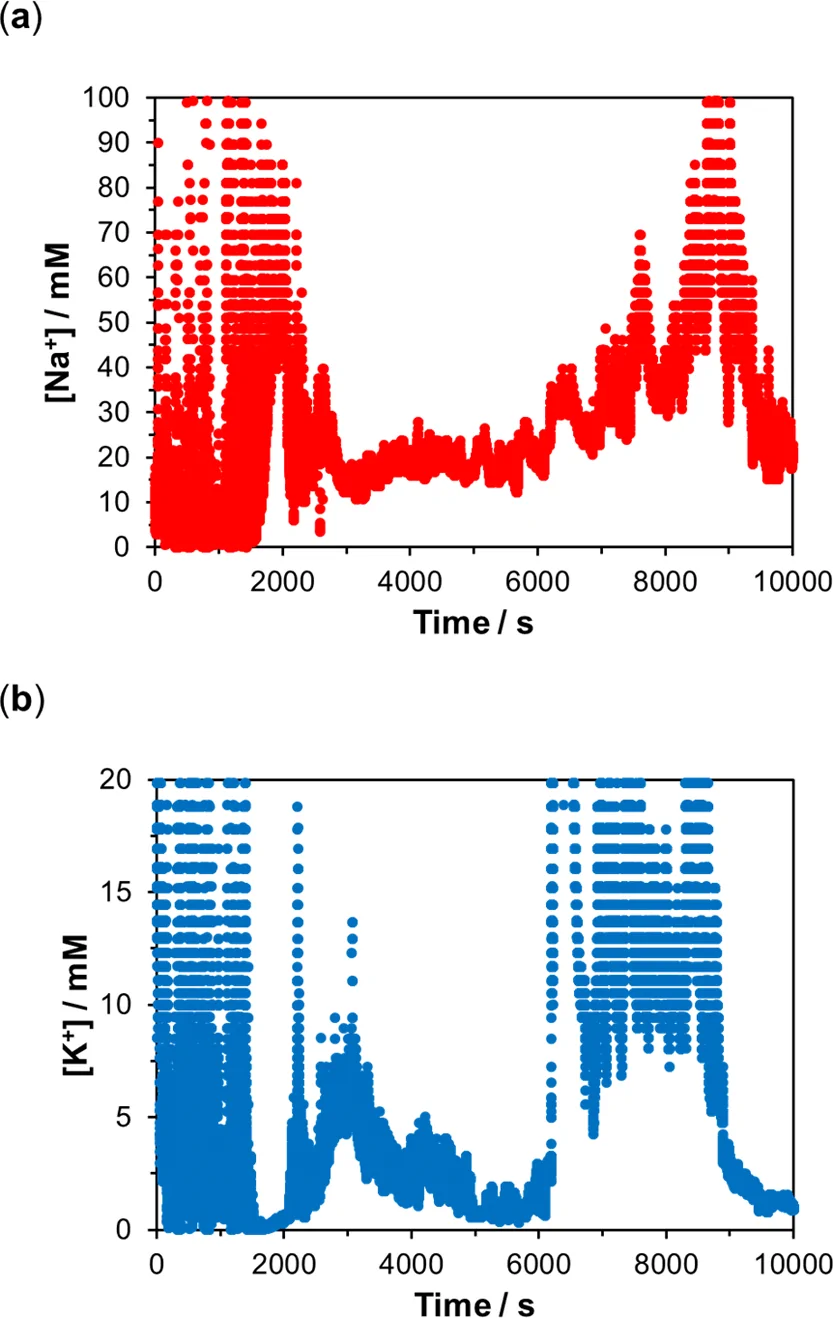
On-body testing results acquired at a construction site. The figure shows the single useable data set obtained from one volunteer construction worker wearing one device. (a) Apparent sodium-ion and (b) apparent potassium-ion concentrations, calculated from the aqueous calibration curves of Figure 3, over approximately 2 h 45 min of construction work. Values exceeding 100 mM for Na^+^ and 20 mM for K^+^ were clipped by the displayed *y*-axis ranges; the concentration conversion itself was not capped. The values are apparent concentrations and are not validated sweat concentrations.

The apparent Na^+^ and K^+^ signals varied over the monitoring period (Figure 8). Because the duration of the on-body measurement (approximately 2 h 45 min) exceeded that of the 3000 s reference-electrode stability test, slow features in the on-body traces may include contributions from reference-electrode drift and from drift of the ion-selective electrodes, neither of which was characterized over the full field duration. Accordingly, the longer-time variations in Figure 8 cannot be attributed solely to temporal changes in the sampled fluid. These values are reported as apparent concentrations from aqueous calibration and are not interpreted physiologically here.

Relating the acquired Na^+^ and K^+^ signals to hydration status or sweat rate, which have been discussed in the physiological literature,^21,22^ would require controlled sweat-rate and hydration measurements and is left to future work. The primary aim of this test was to evaluate whether the wearable sensing device and monitoring system could operate without interrupting the workflow at the construction site.

This test showed that the monitoring system and wearable sensing device can acquire Na^+^- and K^+^-responsive potentiometric signals in real time from a wearer at a large, obstacle-rich location such as a construction site. Because the on-body values are apparent concentrations obtained from aqueous calibration, further studies with controlled hydration protocols and independent sweat sampling are required before the Na^+^ and K^+^ signals can be related to hydration status.

### Comparison with Previous Wearable Ion Sensors

Table 1 compares the present device with representative wearable Na^+^/K^+^ sensors. Relative to these reports, the present contribution is the integration of a printed liquid-junction reference electrode that is operable without prolonged preconditioning, dual Na^+^/K^+^ electrodes, a fabric/SAF sample-handling layer, and a wristwatch-type wireless unit into one device that was operated in the field, rather than the introduction of a new electrode material.

**Table 1:** Comparison of Representative Wearable Sodium- and Potassium-Ion Sensors

study	target ions	fabrication	reference electrode	wireless/field use	distinguishing feature
Gao et al.^13^	Na^+^, K^+^, lactate	sputtering	on-chip	integrated wireless	multiplexed sweat array
Yang et al.^14^	Na^+^, K^+^	screen printing	printed	not reported	cost-effective hydration assessment
Shitanda et al.^15^	Na^+^, K^+^, NH_4_^+^, Cl^–^	screen/transfer printing	printed	not reported	multi-ion heat-transfer printing
this work	Na^+^, K^+^	screen printing	printed liquid-junction; no prolonged preconditioning	wristwatch wireless; construction-site field test	integrated printed liquid-junction RE (no prolonged preconditioning) with fabric/SAF and wireless readout

### Analytical Validation and Scope

The performance parameters evaluated in this study are summarized here and in Table S2. The Na^+^ and K^+^ sensors exhibited sensitivities of 48.3 and 46.4 mV decade^–1^ with linear calibration over 1–1000 mM (*R* ^2^ = 0.998 and 0.997, respectively; Figure 3). Between-electrode reproducibility was assessed using *n* = 3 independently fabricated electrodes for each ion, with error bars in Figures 3 and 4 representing 95% confidence intervals; within-electrode repeatability was not separately determined. The short-term stability of the printed liquid-junction reference electrode was approximately 6 mV over 3000 s (about 7 mV h^–1^) in 10 mM NaCl (*n* = 3), and a functional control without the liquid-junction layer showed a substantially greater dependence on the external KCl concentration (Figure S3). Single-salt cross-response behavior gave apparent concentration-based coefficients of log *K* ^pot^_Na,K_ ≈ – 1.7 and log *K* ^pot^_K,Na_ ≈ – 2.6 (Table S1). Sample exchange through the fabric/SAF configuration produced concentration-dependent responses that tracked the applied NaCl solutions (Figure 5). Field operability was demonstrated over approximately 2 h 45 min of construction work.

The following aspects of quantitative validation remain outside the scope of this field-feasibility study: the analytical accuracy of the on-body apparent concentrations was not established; no independent sweat sampling was performed; no comparison with an established analytical method was made; no pre- or postfield calibration was performed; the long-term stability of the complete Na^+^/K^+^ sensing system was not evaluated; and no validation under a realistic mixed-ion sweat background was performed.

### Limitations

The main limitations of this study are as follows. The on-body values in Figure 8 are apparent concentrations from aqueous calibration under conditions that differ from the on-body environment and are not validated sweat concentrations. A known-concentration recovery test through the complete fabric/SAF structure was not performed. The printed reference electrode drifted by about 6 mV over 3000 s, which is small but not negligible relative to the sensor sensitivity, and the long-term stability of the complete sensing system was not evaluated. Selectivity was assessed only in single-salt solutions without activity correction. The wristwatch-circuit and potentiostat traces were compared only qualitatively. A control reference electrode without the liquid-junction layer was evaluated, but only in 1 and 100 mM KCl (Figure S3); it was not evaluated across the NaCl concentration series used for Figure 2, and a condition-matched quantitative comparison between the two electrode types was therefore not performed. The membrane composition was not optimized for the heat-pressed format. The apparent selectivity coefficients were calculated using the theoretical Nernstian slope, and no mixed-ion validation was performed. Limits of detection and quantification were not determined because blank-response variability was not evaluated.

## Conclusion

We built a wristwatch-type dual-ion wearable device that incorporates a previously reported screen-printed liquid-junction reference-electrode architecture together with Na^+^- and K^+^-selective electrodes, a fabric/SAF sample-handling layer, and a custom wireless unit. On the bench, the sensors showed sensitivities of 48.3 and 46.4 mV decade^–1^ for Na^+^ and K^+^, the printed reference electrode operated without prolonged preconditioning and showed a drift of about 6 mV over 3000 s, and single-salt cross-response behavior was evaluated. A control reference electrode fabricated without the liquid-junction layer showed a substantially greater dependence on the external KCl concentration together with continued time-dependent potential changes, supporting the functional role of the liquid-junction layer. In the field, the device operated at an active construction site and acquired Na^+^- and K^+^-responsive potentiometric signals in real time; these were converted to apparent concentrations using aqueous calibration curves. Establishing quantitative sweat analysis will require a recovery test through the complete fabric/SAF structure, selectivity measurements under a realistic sweat background, and validation against controlled sweat collection and hydration protocols.

## Supplementary Material



## Notes

Figure S1, evaluation of a PVB-type printed reference electrode in 0.1 M NaCl; Figure S2, raw and enlarged wristwatch-circuit and potentiostat traces in 1 mM NaCl; Figure S3, evaluation of a printed reference electrode fabricated without the liquid-junction layer in 1 and 100 mM KCl; Table S1, separate-solution potentials and apparent cross-response coefficient estimates; Table S2, summary of evaluated performance parameters and remaining validation limitations; Text S1, on-body testing protocol (PDF)

## ⊥

Graduate School of Information Science and Technology, Hokkaido University, Kita 14, Nishi 9, Kita-ku, Sapporo, Hokkaido 060–0814, Japan

## #

IHI Corporation, Yokohama Office (Engineering Center), 1, Shin-Nakahara-cho, Isogo-ku, Yokohama-shi, Kanagawa 235–8501, Japan.
