# Supplementary material for: Real-Time Monitoring with a Wristwatch-Type Sodium- and Potassium-Ion Sensor Using a Screen-Printed Liquid-Junction Reference Electrode
Source: ACS Omega. 2026 Sep 21;11(39):59011–20. doi: 10.1021/acsomega.6c04240 (PMC13647336; doi:10.1021/acsomega.6c04240)
Supplement: Supplementary file 1 [file ao6c04240_si_001.pdf]

Supporting Information

Real-Time Monitoring with a Wristwatch-Type Sodium- and Potassium-Ion Sensor Using a Screen-Printed Liquid-Junction Reference Electrode

Isao Shitanda,[1,2,‡,*] Takuma Fujisawa,[1,‡] Noya Loew,[1,§] Ryo Shirai,[3] Yuta Imade,[1] Yuri Okamoto,[1,¶] Shinya Yanagita,[2,4] Hikari Watanabe,[1] and Masayuki Itagaki[1,2]

[1] Department of Pure and Applied Chemistry, Faculty of Science and Technology, Tokyo University of Science, 2641 Yamazaki, Noda, Chiba 278-8510, Japan
[2] Research Institute for Science and Technology, Tokyo University of Science, 2641 Yamazaki, Noda, Chiba 278-8510, Japan
[3] Department of Informatics, Kyoto University, 36 Yoshida-honmachi, Sakyo-ku, Kyoto 606-8501, Japan
[4] Institute of Arts and Sciences, Tokyo University of Science, 2641 Yamazaki, Noda, Chiba 278-8510, Japan
*Corresponding author: Isao Shitanda (shitanda@rs.tus.ac.jp)
‡These authors contributed equally.
§Present Address: Present Address: Graduate School of Information Science and Technology, Hokkaido University, Kita 14, Nishi 9, Kita-ku, Sapporo, Hokkaido 060-0814, Japan.
¶Present Address: IHI Corporation, Yokohama Office (Engineering Center), 1, Shin-Nakahara-cho, Isogo-ku, Yokohama-shi, Kanagawa 235-8501, Japan.

This file contains Figures S1–S3, Tables S1 and S2, and Text S1.

Figure S1

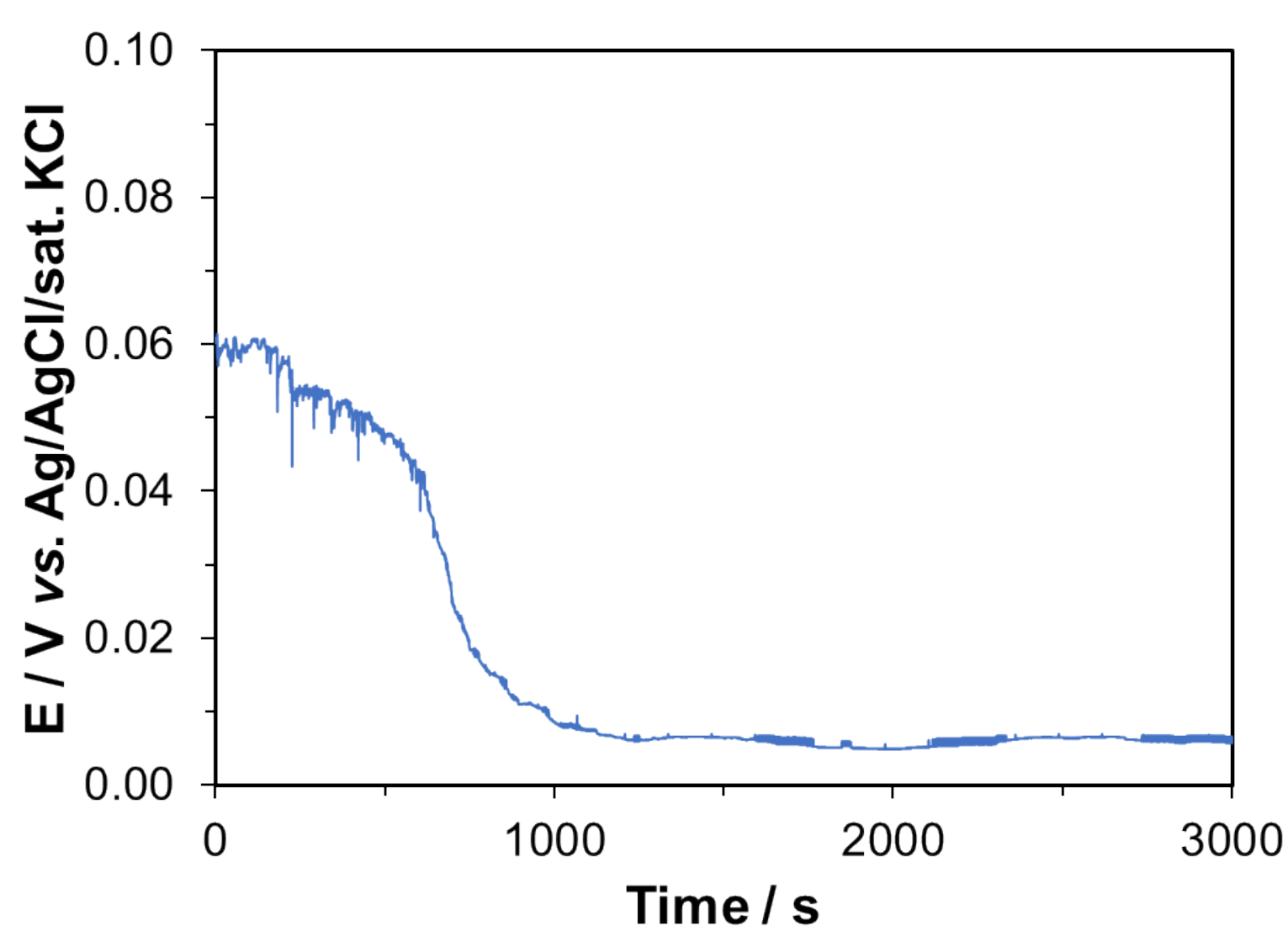

Figure S1. Evaluation of a PVB-type reference electrode. Potential difference between the printed PVB-type reference electrode and a commercial Ag/AgCl/saturated-KCl reference electrode as a function of time. Measurements began immediately following immersion of the printed reference electrode in 0.1 M

aqueous NaCl. The measurement was made on the printed strip without the separate superabsorbent-fiber layer.

Figure S2

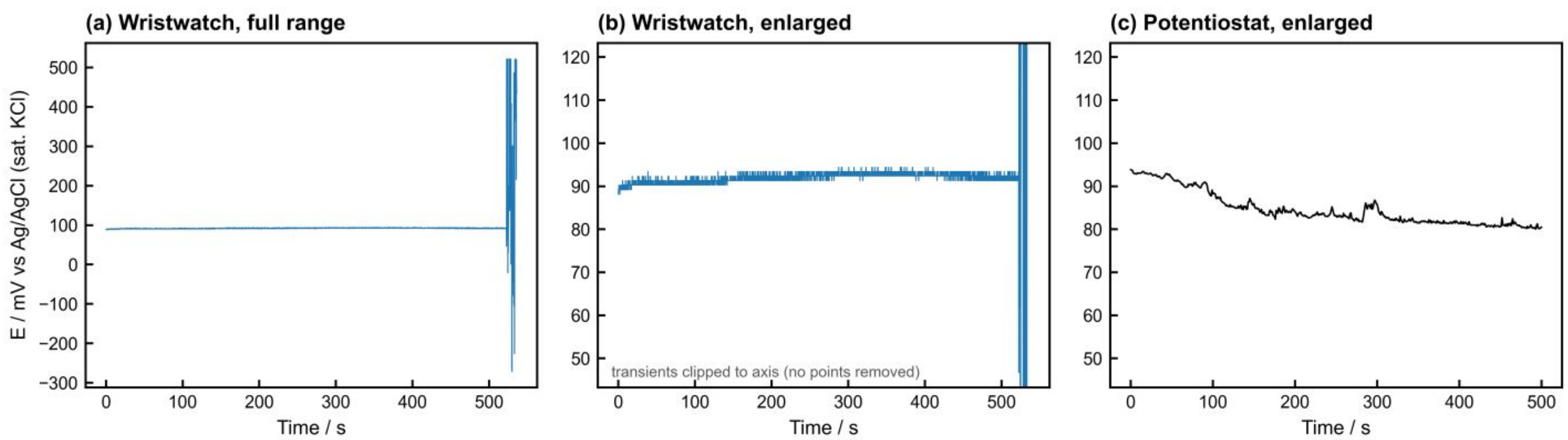

Figure S2. Open-circuit potential of the sodium-ion sensor in 1 mM NaCl recorded with the wristwatch circuit and with a commercial potentiostat (raw data; no points removed). (a) Complete wristwatch-circuit trace over the full potential range, including intermittent transients presumed to be associated with wireless operation. (b) The same wristwatch-circuit trace on a restricted potential axis so that the baseline is legible; the transients extend beyond the axis and are clipped for display, but no data points were removed. (c) Potentiostat trace on the same restricted axis. The comparison is qualitative; the two traces were recorded separately. The wristwatch-circuit trace was sampled at 10 samples s^{-1}, and the potentiostat trace is shown at its native sampling rate.

Figure S3

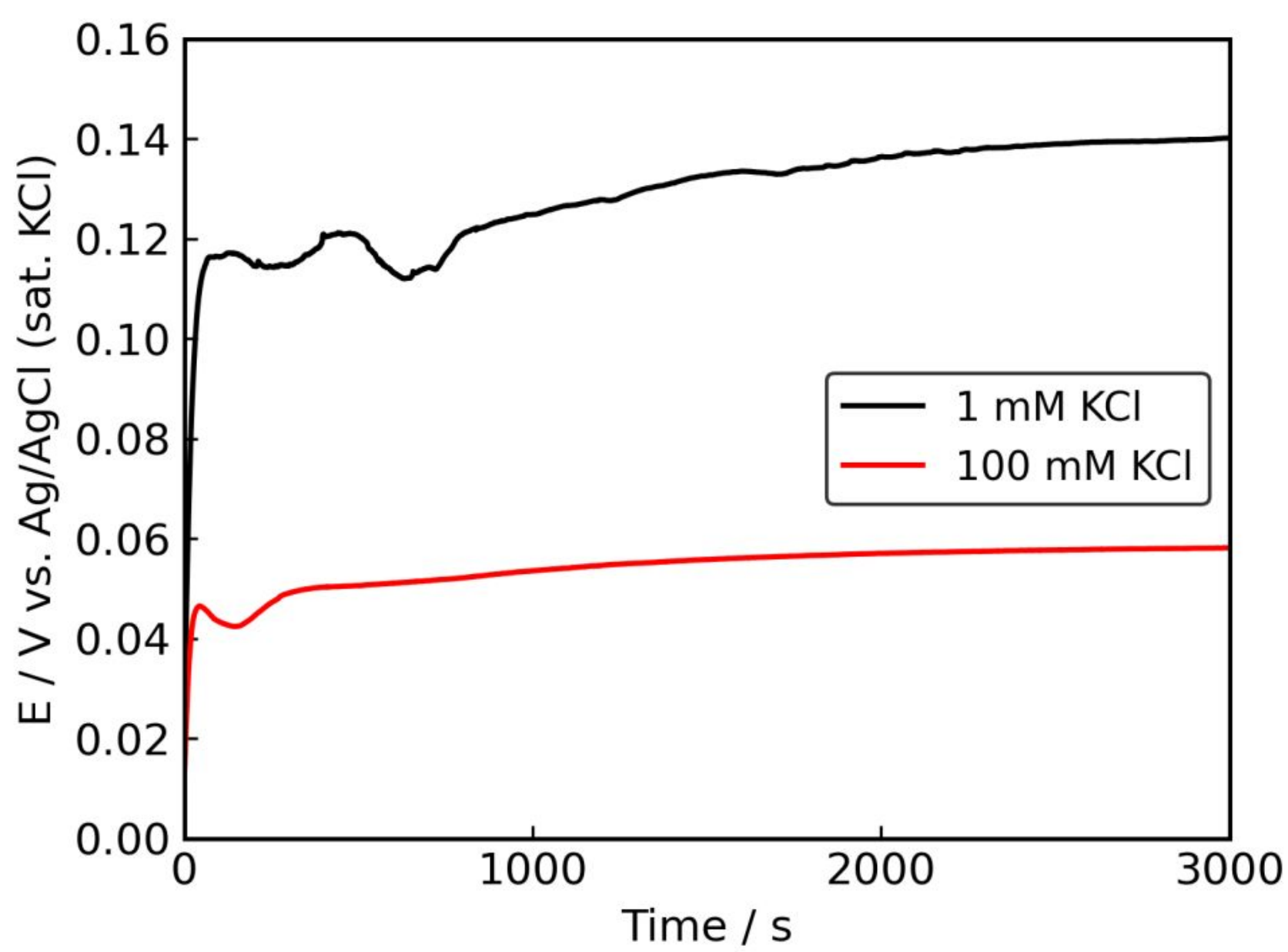

Figure S3. Evaluation of a printed reference electrode fabricated without the liquid-junction layer. Potential of the control electrode measured against a commercial Ag/AgCl/saturated-KCl reference electrode in 1 mM (black) and 100 mM (red) aqueous KCl. Measurements began after immersion of the electrode in the test solution and continued for 3000 s. The potential depended strongly on the external KCl concentration, with a difference of approximately 80 mV between 1 and 100 mM KCl at 3000 s, and continued to change with time over the measurement period. The control electrode was fabricated using the same process as the liquid-junction reference electrode, including the KCl-containing electrolyte layer, except that the liquid-junction layer was omitted. A single control electrode was evaluated sequentially in each KCl concentration (n = 1).

Table S1

Apparent concentration-based separate-solution coefficients were estimated from the open-circuit potentials at 10 mM against the commercial Ag/AgCl/saturated-KCl reference electrode, using the Nicolsky–Eisenman relation for monovalent ions, $\log K = (E_{interfering} - E_{primary}) / S$, with $S = 59.2$ mV decade^{-1} at 25 °C (theoretical Nernstian slope).[1] Because the measured primary-ion slopes were sub-Nernstian (48.3 and 46.4 mV decade^{-1}), these are apparent coefficients calculated under the theoretical Nernstian-slope assumption and provide an approximate cross-response index rather than rigorous thermodynamic selectivity coefficients. Potentials are concentration-based (no activity correction); n = 3 independently fabricated electrodes. Under the tested single-salt conditions, the K^+-selective electrode showed a substantially smaller response to NaCl than to KCl, whereas the Na^+-selective electrode showed an appreciable response to KCl.

**Quantity**	**Na^+ sensor**	**K^+ sensor**
E in primary salt at 10 mM (mV)	138.4 (NaCl)	203.2 (KCl)
E in interfering salt at 10 mM (mV)	39.1 (KCl)	50.2 (NaCl)
Apparent log K^{pot}	−1.7	−2.6
Apparent K^{pot}	≈ 0.02	≈ 0.003

Reference: (1) Umezawa, Y.; Bühlmann, P.; Umezawa, K.; Tohda, K.; Amemiya, S. Potentiometric Selectivity Coefficients of Ion-Selective Electrodes. Part I. Inorganic Cations (IUPAC Technical Report). Pure Appl. Chem. 2000, 72, 1851–2082.

Table S2

Table S2. Summary of evaluated performance parameters and remaining validation limitations of the Na^+/K^+ sensing system. Table S2 summarizes the performance parameters evaluated in this study together with the aspects of quantitative validation that remain outside the scope of the present field-feasibility demonstration. Parameters listed as “not established” or “not performed” were not measured in this study.

**Parameter**	**Na^+ sensor / system**	**K^+ sensor / system**	**Source / qualification**

Sensitivity	48.3 mV $decade^{-1}$	46.4 mV $decade^{-1}$	Figure 3; 1–1000 mM; vs commercial Ag/AgCl/sat. KCl
Linearity	$R^2 = 0.998$	$R^2 = 0.997$	Figure 3
Between-electrode reproducibility	n = 3	n = 3	Independently fabricated electrodes; error bars are 95% confidence intervals; within-electrode repeatability not separately determined
Cross-response (apparent, single-salt)	log $K^{pot} \approx -1.7$ ($K^{pot} \approx$ 0.02) against K^+	log $K^{pot} \approx -2.6$ ($K^{pot} \approx$ 0.003) against Na^+	Table S1; concentration-based, theoretical Nernstian slope, no activity correction
Reference-electrode short-term stability	approximately 6 mV over 3000 s (about 7 mV h^{-1})	same reference electrode	10 mM NaCl; n = 3
No-liquid-junction control	approximately 80-mV dependence on external KCl concentration (1 vs 100 mM at 3000 s), with continued time-dependent change	same control electrode	Figure S3; KCl only; not condition-matched to Figure 2
Sample exchange (fabric/SAF)	Concentration-dependent response to stepwise NaCl delivery	Not independently tested	Figure 5; not a known-concentration recovery test
Field-operation duration	approximately 2 h 45 min	approximately 2 h 45 min	Feasibility only; not analytical validation
Accuracy vs established analytical method	Not established	Not established	Limitation
Independent sweat sampling	Not performed	Not performed	Limitation
Long-term stability of complete system	Not evaluated	Not evaluated	Limitation
LOD / LOQ	Not determined	Not determined	Blank-response variability not evaluated

Text S1. On-body testing protocol

On-body testing was carried out at an active construction site managed by Sanwa Kensetsu Co., Ltd. One construction-worker volunteer wore one wristwatch-type wireless sensing device while performing the assigned tasks according to the usual routine, including breaks, hydration, and meals, over a working period of approximately 2 h 45 min; one usable dataset was obtained. The recorded potentials were converted to apparent Na^+ and K^+ concentrations using the aqueous calibration curves of Figure 3 in the main text; no independent sweat sampling and no pre- or post-field calibration were performed. Testing was conducted in accordance with the ethical guidelines approved through the "Medical Research Plan Involving Human Subjects" review process of the Tokyo University of Science, and the volunteer participated after being informed of the procedure.
